# Effect of Eco-Friendly Peanut Shell Powder on the Chemical Resistance, Physical, Thermal, and Thermomechanical Properties of Unsaturated Polyester Resin Composites

**DOI:** 10.3390/polym13213690

**Published:** 2021-10-26

**Authors:** Przemysław Pączkowski, Andrzej Puszka, Barbara Gawdzik

**Affiliations:** Department of Polymer Chemistry, Institute of Chemical Sciences, Faculty of Chemistry, Maria Curie-Sklodowska University in Lublin, Gliniana 33, 20-614 Lublin, Poland; andrzej.puszka@umcs.pl (A.P.); barbara.gawdzik@umcs.pl (B.G.)

**Keywords:** unsaturated polyester resin, eco-friendly, biofiller, peanut shell, lignocellulosic biomass, composites, immersion test, chemical resistance

## Abstract

The paper investigates the synthesis of eco-friendly composites and their properties before and after immersion in solvents of different chemical natures. For their preparation, unsaturated polyester resin (UPR) based on recycled poly (ethylene terephthalate) (PET) and peanut shell powder (PSP) were used. Polymerization was carried out in the presence of environmentally friendly polymeric cobalt. Distilled water, acetone, 10% hydrochloric acid, 40% sodium hydroxide, toluene, and 2% sodium carbonate were used as solvents in the chemical resistance test. Changes in the structure, properties, and appearance (morphology) of composites after 140 days of immersion in solvents were investigated. The results show that both the resin and its composites show resistance towards 10% HCl and toluene. The immersion in water has no significant effect on the resin, but for PSP composites, the plasticizing effect of water was observed. In acetone, after only one day, the resin and its composite with 10% PSP shrink and fall into pieces. However, the most destructive is an alkaline environment. After the immersion test, a huge increase in mass and a deterioration of gloss and thermomechanical properties were observed. The destructive influence of the 40% NaOH environment mainly concerned the resin.

## 1. Introduction

Due to their excellent properties, polymer composites are used in a variety of industrial and household applications. The quality of composites depends on the selection of the constituent materials. In recent years, the most frequently exploited fillings for composites were glass or carbon fibers [1,2,3]. To minimize the environmental pollution due to the use of synthetic composites, scientists’ present focus is on natural fillers. The properties of such biocomposites are not worse than those of the polymers constituting the matrix and, moreover, they have a greater chance to decompose in the natural environment. Compared to composites filled with glass and carbon fibers, they have undoubtedly lower thermal and chemical resistance and less stable mechanical properties over time, but they can be effectively used for immediate purposes [4,5,6,7,8].

Natural fibers are generally classified into different types depending on their origin. Animal fibers, such as hair and silk, and mineral fibers have not been widely used as reinforcement fibers. Natural plant fibers include different classes of fibers such as bast (flax, hemp, jute, kenaf, ramie), seed (coir, cotton, kapok), straw (corn, rice, wheat), grass (bamboo, bagasse), leaf (abaca, banana, curaua, henequen, pineapple, sisal), and wood fibers (softwood, hardwood). The major chemical composition of plant fibers is lignocellulose (cellulose, hemicellulose, and lignin) and the amount of these components depend on the fiber type [9,10,11,12].

The most common natural filler is wood fiber or flour, which is a waste product in the production of paper [13,14,15]. However, waste rice husks, nut shells, stalks of hemp, sisal, jute, or corn cobs are being used more often [16,17,18]. These natural ingredients reduce the polymer product costs, and on the other hand, they are characterized by good reinforcing properties and biodegradability.

In our previous papers, the properties of the composites based on wood flour and unsaturated polyester resin were studied [19,20]. Unsaturated polyester resins are the most widely used thermoset matrices in polymeric composites. They are attractive because of their low cost, ease of processing, capability of being cured at room temperature, and availability. Their mechanical, chemical, thermal, and electrical properties meet most of the requirements necessary for the production of large-scale products, such as laminates, bulky industrial constructions, insulations, and molding compounds. In this paper, the commercially available unsaturated polyester resin was used for the production of composites with peanut shell powder as a natural filler.

The peanut is one of the most important food crops cultivated in countries with a subtropical climate and is a valuable foodstuff used all over the world. Peanuts are edible but their shells are waste. The peanut shell is a lignocellulosic material composed of cellulose (44.8%), hemicellulose (5.6%), and lignin (36.1%) with a complex fibrous structure [21]. Of note is the large content of lignin in the fibers, much higher than in coconut, hemp, or sisal, which can be exploited for the improvement of composite reinforcing properties [22]. For example, Obasi [23] reported that the addition of peanut filler to polyethylene reduced the tensile strength and elongation at break, whereas compositions obtained with maleate polyethylene as a compatibilizer improved these properties. Zaaba et al. [24] incorporated peanut shell powder into recycled polypropylene. To decrease the water absorption and tensile modulus of the obtained composites, a compatibilizer was added. Yamoum and Magaraphan [25] studied the effect of peanut flour content on mechanical, thermal, and biodegradable properties of composites with polylactic acid. The scientific literature also includes reports on composites of peanut flour with thermoset polymers. The composites of epoxy resins with the peanut flour showed better mechanical and thermal properties compared to those of pure resins [26]. The peanut powder was also applied in the preparation of composites with unsaturated polyester resin. Ilkadious et al. [27] studied the influence of virgin and modified peanut shell powder on water uptake as well as the thermal and mechanical properties of the obtained composites.

In this paper, the chemical resistance of the composites containing different amounts of peanut filler is studied. The motivation for these studies was to learn about the performance properties of composites subjected to unfavorable environmental conditions, such as water, 10% HCl, 2% Na_2_CO_3_, 40% NaOH, acetone, and toluene. The influence of solvents on the structure, thermal, and thermomechanical properties and texture of pure resin and its PSP composites was investigated.

## 2. Materials and Methods

### 2.1. Chemicals

The unsaturated orthophthalic/styrene mixture resin from recycled PET, Estromal 14PB-06 NZ (acidic value 13.4 mg KOH g^−1^, viscosity at 23 °C 356 mPas, non-volatile content 61.2 wt.%, and reactivity factor 1.53) was provided by LERG (Pustków, Poland). Methyl ethyl ketone peroxide (Luperox DHD-9) as an initiator was provided by Sigma-Aldrich (St. Louis, MO, USA). Furthermore, a 4% polymeric cobalt solution used as an accelerator was synthesized in the Department of Polymer Chemistry, Institute of Chemical Sciences, Maria Curie-Sklodowska University in Lublin (Lublin, Poland).

The Virginia-type peanut shells collected from the Southeastern USA were ground in an analytical mill A11 basic from IKA Werke GmbH & Co. KG (Staufen, Germany). The obtained powder was separated using a sieve shaker and then dried at 80 °C for 2 h before use. For the UPR/PSP composites’ preparation, peanut shell powder of a sieve fraction of 0.10–0.25 mm was applied.

### 2.2. Preparation of Peanut Shell Powder Composites

The polymeric composites were prepared by mixing the UPR with varying weight percentages of peanut shell powder up to 30. Based on the UPfR content, 1.1 wt.% methyl ethyl ketone peroxide (Luperox DHD-9) was added as an initiator and 0.25 wt.% of 4% polymeric cobalt solution was added as a promoter. The mixture was well stirred until homogeneity was reached and then poured into the cuboid-shape mold. The prepared composites were allowed to be cured at room temperature for 24 h and then post-cured in the oven at 80 °C for 10 h. The compositions of the prepared materials are presented in Table 1.

### 2.3. Specimen Preparation

In order to study the properties of the UPR/PSP composites, their samples were prepared in an appropriate manner. The cuboid composite samples sized 65 mm × 10 mm × 4 mm were cut with the MFG 8037P CNC milling machine from Ergwind (Gdańsk, Poland). The samples were immersed in different solvents. An additional sample that was not used in the chemical resistance test was used as the reference sample.

### 2.4. Research Methods

#### 2.4.1. Elemental Analysis of Peanut Shells

The elemental analysis of peanut shell powder was conducted using a CHNS-analyzer EuroEA3000 from EuroVector (Pavia, Italy). The device allowed for simultaneous determination of the percentage of carbon (%C), hydrogen (%H), nitrogen (%N), and sulfur (%S). The oxygen content (%O) is determined using Equation (1) [28]:

(1) $$\%O=100\%-\left(\%C+\%H+\%N+\%S\right)$$

#### 2.4.2. Chemical Resistance/Immersion Test

The behavior of the UPR/PSP composites in the presence of chemical liquids was determined according to the EN ISO 175: 2010 [29] standard. The samples with the dimensions 65 mm × 10 mm × 4 mm were immersed separately in the airtight containers containing 50 mL of the tested liquid. They were placed in the dark at room temperature (23 °C ± 2 °C). The chemical resistance of the samples was tested in distilled water, 10% HCl, 2% Na_2_CO_3_, 40% NaOH, acetone, and toluene. Periodically, the samples were removed from the solvents, rinsed with distilled water, and wiped gently. The mass change Δ*m* is determined using Equation (2) [29]:

(2) $$\Delta m=\frac{m_{i}-m_{0}}{m_{0}}\times 100$$

where:

*m*_0_ is the initial mass of the specimen.
*m_i_* is the mass of the specimen after immersion.

#### 2.4.3. FT-IR Spectroscopy Analysis

The functional groups and chemical characteristics of peanut shell powder and their composites were analyzed by Fourier transform infrared spectroscopy (FT-IR) using a Bruker TENSOR 27 spectrometer (Ettlingen, Germany) with a resolution of 4 cm^−1^ in a frequency range from 600 to 4000 cm^−1^ at 32 scans per sample. The analysis was preceded by the background spectrum measurements.

#### 2.4.4. Thermal and Thermomechanical Properties

The thermal stability of the peanut shell powder and the UPR/PSP composites were evaluated by thermogravimetry (TG) and differential thermogravimetry (DTG) characterization. The TG scans were collected by the Netzsch Simultaneous Thermal Analyzer STA 449F5 Jupiter (Selb, Germany) from 30 to 1000 °C at a heating rate of 10 °C min^−1^ in the oxidative atmosphere (air).

To determine the thermomechanical properties of the UPR/PSP composites, the dynamic mechanical analyzer (DMA) Q800 from TA Instruments (New Castle, DE, USA), equipped with a dual-cantilever device, was used. The temperature scanning was carried out from −50 °C to 200 °C with a constant heating rate of 3 °C min^−1^ at a sinusoidal distortion of 10 µm amplitude and a 1 Hz frequency. The samples (65 mm × 10 mm × 4 mm) were tested before and after the immersion in liquid chemicals. The test procedure was in accordance with the standard EN ISO 6721-1:2019 [30]. The glass-transition temperature (T_g_), mechanical loss factor (tan δ), values of storage modulus (E’), and Full Width at Half Maximum (FWHM) were determined.

#### 2.4.5. Surface Analysis and Morphology Studies

To determine the morphology, structure, and texture of peanut shells, micrographs of the sample were taken using the FEI QUANTA 3D FEG high-resolution scanning electron microscope (Hillsboro, OR, USA) at an acceleration voltage of 5 kV. The sample was covered with a thin layer of Pd/Au to avoid electrostatic charging during the examination.

The gloss measurement was made using the triple-angle gloss meter, Zehntner ZGM 1110 from Zehntner GmbH Testing Instruments (Sissach, Switzerland). This device operates simultaneously in one of three geometric units in which the values of angles, 20°, 60°, and 85°, are related to a high gloss or matte surface: Standard Gloss: 20° (86.8 GU), 60° (93.4 GU), and 85° (99.7 GU). These determinations were made according to the standard ASTM D2457 [31]. The final result was the arithmetic averaging of ten measurements before and after immersion in liquid chemicals.

The changes on the surface of the samples after the immersion test were determined on the basis of photos taken with the Morphologi G3 apparatus from Malvern Instruments Limited (Malvern, UK).

#### 2.4.6. Statistical Data Evaluation

All data were subjected to the analysis of variance using the Origin 8.5.0 (OriginLab, Northampton, MA, USA) applications. The one-way analysis of variance (one-way ANOVA) was used to detect significant differences among the gloss measurement depending on the PSP content.

## 3. Results and Discussion

### 3.1. Peanut Shell Powder Analysis

The assessment of the suitability of the PSP for the preparation of composites should begin with the characteristics of the filler itself. The results of the CHNS analysis show that the peanut shell powder is composed of 46.05% carbon, 5.76% hydrogen, 1.37% nitrogen, and 46.82% oxygen. The oxygen content in the filler was determined using Equation (1). These are the elements that build cellulose, hemicellulose, lignin, and proteins, the main components of the shell.

**Figure 1:**
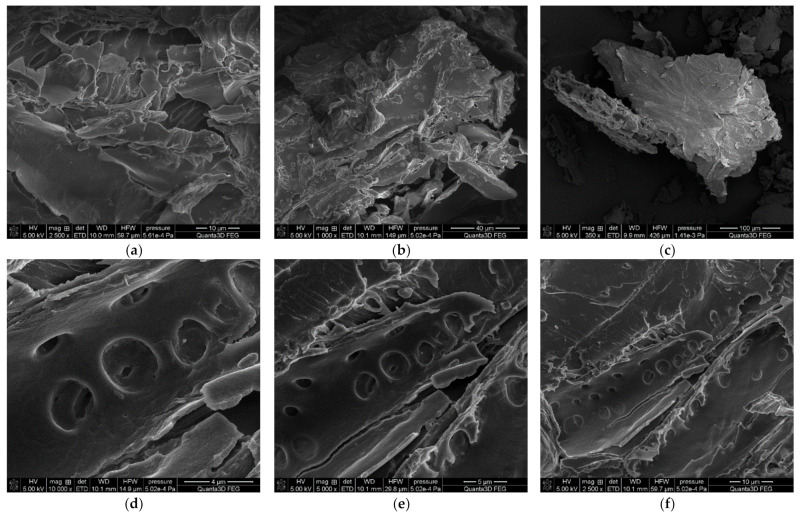
Scanning electron micrographs of the peanut shell powder; (**a**–**c**) SEM images of peanuts shell powder (magnification 350–2500×). (**d**–**f**) peanuts shell powder (magnification 2500–10,000×).

The morphological analysis of the peanut shell powder was performed using scanning electron microscopy and is shown at different magnifications in Figure 1. The shell powder of the peanuts showed fine sheet- or plate-like structures and aggregates with sharp edges (Figure 1a–c). At a high magnification of the SEM micrograph, simple pitted cell walls with caves or pore structures were observed (Figure 1d–f).

**Figure 2:**
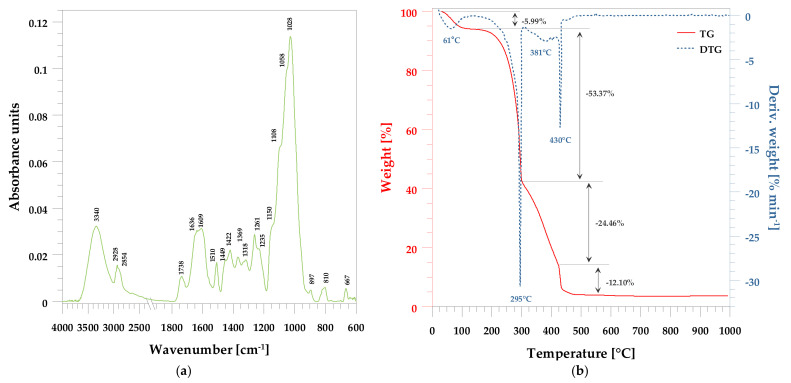
Peanut shell powder analysis. (**a**) FT-IR spectrum of the PSP; (**b**) thermal decomposition of the PSP.

Figure 2a shows the FT-IR/ATR spectrum of the peanut shell powder. As expected, the spectrum for this lignocellulosic material is extremely complicated. It is caused by multiple various functional groups involved in the molecules of cellulose, hemicellulose, and lignin. This biofiller offers many functional groups, such as carboxyl, carbonyl, hydroxyl, and amino, in characteristic chemical structures.

The characteristic vibration bands in the FT-IR/ATR spectrum (Figure 2a) of the peanut shell powder are shown in Table 2. The assignment of the bands is consistent with those given in many articles dealing with lignocellulosic material [10,22,27,32].

TG and DTG thermal stability determinations were also made for the PSP (Figure 2b). From the course of the TG and DTG curves. one can see that the initial weight loss starts at rather low temperatures. Although the shells were dried, moisture and certain volatile compounds evaporate at temperatures 40–100 °C.

Real degradation takes place in the other stages. As shown in Figure 2b, the TG curve of peanut shell powder shows a significant loss in the temperature range of 190–500 °C. On the DTA curve, two peaks are visible at 295 and 430 °C. They could be attributed to the degradation of the hemicellulose (around 300 °C), cellulose (around 380 °C), and lignin (around from 200 °C to 500 °C).

The temperature range of 200 to 380 °C essentially corresponds to the cellulosic chain degradation including several processes such as the depolymerization, dehydration, and decomposition of glycosidic units. Above 380 °C, the decomposition of carbonic residue into low-molecular-weight components pccurs.

### 3.2. UPR/PSP Composites Analysis

**Figure 3:**
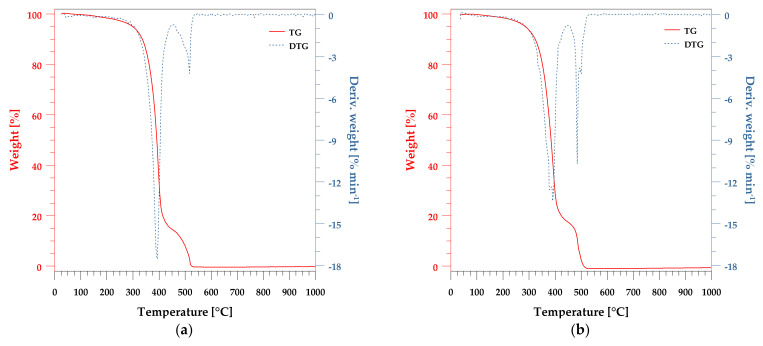

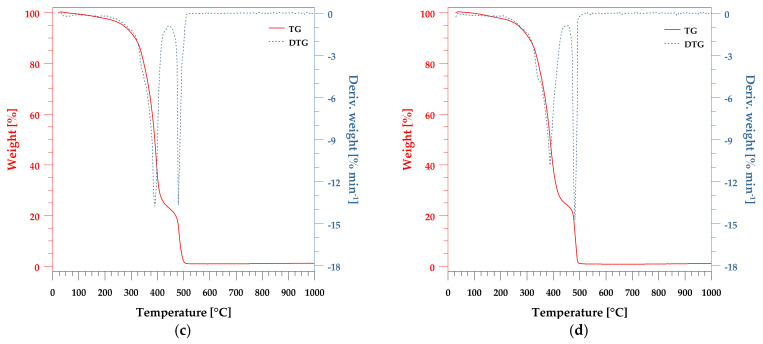
Thermal decomposition of the UPR/PSP composites. TG and DTG curves of (**a**) pure UPR; (**b**) UPR + 10; (**c**) UPR + 20, (**d**) UPR + 30.

Table 3 and Figure 3 show the results of the thermal analysis of pure unsaturated polyester resin, peanut shell powder, and reinforced UPR/PSP composites. Thermal properties are an important characteristic that should be considered in order to evaluate the overall performance of PSP-reinforced polymer composites. Based on the TG and DTG curves, it can be seen that the pure resin has two degradation steps with a decomposition maximum at 394 °C and 519 °C. These maximum decomposition temperatures are much higher than those for the filler (61, 295, 381, 430 °C).

In the case of UPR/PSP composites, two stages of degradation are observed. The curves are almost analogous to the pure resin, but for the composite containing 10% filler, the second peak is split into two at 480 °C and 519 °C. For UPR + 20 and UPR + 30 composites, the peak at 519 °C disappears and only the peak at 480 °C remains. As the amount of filler increases, it can be seen that most of the degradation takes place in the second region.

For the composites, degradation includes the thermal decomposition of cellulose, hemi-cellulose, and lignin as well as the polymer matrix. The weight change decreased in the first decomposition area from 85.71 to 75.42%, and in the second, it increased from 14.19 to 23.52%. The amount of filler also had an effect on the residual mass. For the composite with the 10% content, it was 0.65%, and it increased to 1.22% for that with the PSP content while for the pure resin it was 0.

At the same time, an increase in the amount of filler in the composite resulted in a decrease in the characteristic temperatures. The onset of the decomposition temperature T_1%_ for the resin was 167 °C and gradually decreased to 142 °C as the PSP content increased. For the values of temperature T_5%_ mass loss, a similar tendency can be observed. Only the temperatures of 50% mass loss are independent of the PSP content.

As the obtained materials were subjected to the chemical resistance test, it was necessary to examine what influence it had on the structure and properties. The spectroscopic analysis of the unsaturated polyester resin was discussed earlier [19]. The strong absorption bands at 1719, 1260, and 1124 cm^−1^ correspond to the vibrations of the carbonyl and ester groups, respectively, while 700 cm^−1^ is due to the aromatic ring.

**Figure 4:**
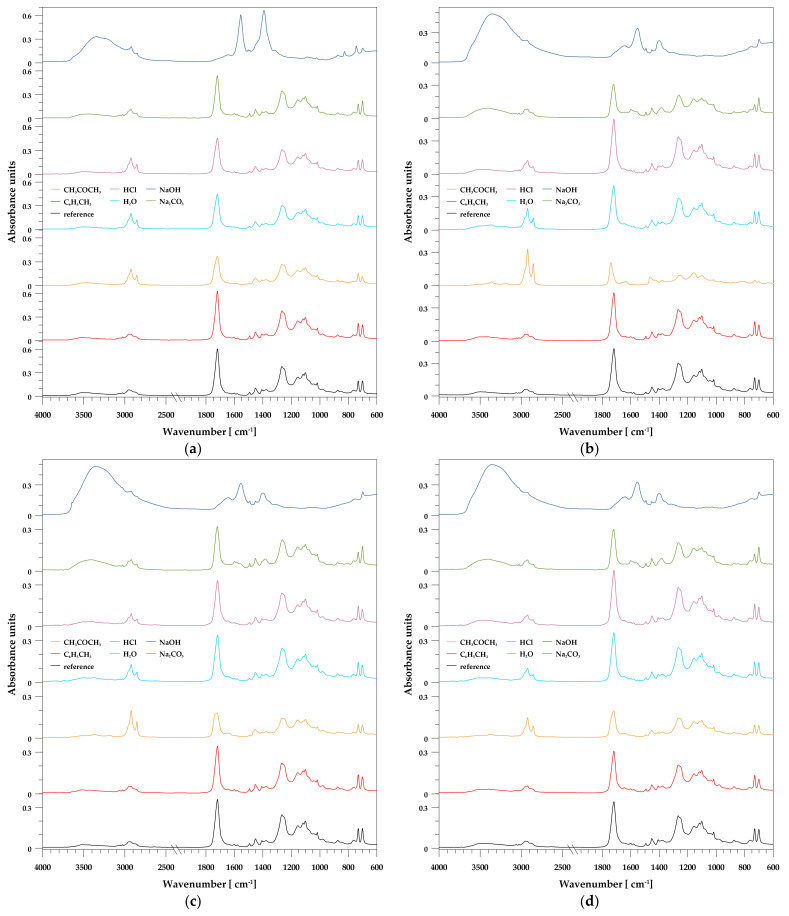
FT-IR/ATR spectra of the UPR/PSP composites before and after the immersion test. (**a**) Pure UPR; (**b**) UPR + 10; (**c**) UPR + 20; (**d**) UPR + 30.

In Figure 4, the spectra of the UPR/PSP composites immersed in different chemicals are presented. After the immersion test in toluene, no spectral changes are observed. This solvent does not alter the chemical structure of the materials, neither the polymer matrix nor the filler. For the sample of unsaturated polyester resin, the band at 700 cm^−1^ is representative of styrene-benzene rings (aromatic -CH ring) and does not change during immersion because styrene cannot be hydrolyzed [33].

Interesting spectral changes are observed for the samples immersed in acetone. For pure resin and its composites with PSP, the most important changes concern the intensity of the absorption bands. A significant increase is observed at about 2950 cm^−1^, which is responsible for the vibrations of the methylene groups, while the intensity of the characteristic vibration band of the carbonyl and the ester groups decreases. A similar observation regarding the UPR was made by Abellache et al. [34]. Another effect observed in the infrared spectra of immersed composites is the shifting and broadening of the carbonyl band.

For the samples immersed in distilled water, a broad band corresponding to the OH group vibrations of about 3600 to 2800 cm^−1^ is observed. This indicates the phenomenon of hydrolysis or water absorption by the resin and filler. This is also true of samples exposed to aqueous solutions. However, the mentioned effect is most visible in the case of the sample immersed in alkaline solutions (NaOH, Na_2_CO_3_). An increase in the intensity of the methylene group band is also observed. Signals from both the ester and ester carbonyl stretching vibrations are reduced. This result suggests that the breakage of the ester bond occurred due to hydrolytic degradation. Similar observations regarding the UPR were made by Visco et al. [35].

The spectra of composites immersed in NaOH are difficult to unambiguously inter-pret. This is due to the absorption of a large amount of liquid into the structure of the materials and the fact that the vibration bands come from an alkaline solution.

In the polymer composite, the water transport can be facilitated in varied pathways, such as inside the matrix, the imperfections within the matrix (microspace, pores, or cracks), and by the capillarity along the fiber–matrix interface.

**Figure 5:**
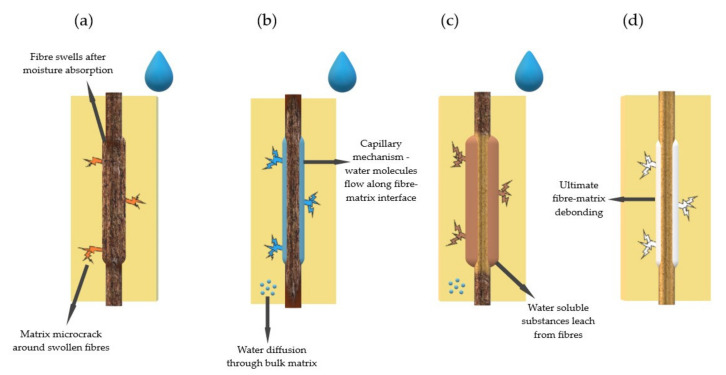
Degradation during the immersion test. Effect of water penetration into the fiber–matrix composite: (**a**) Moisture sorption; (**b**) capillary mechanism; (**c**) substance leaching from fiber; (**d**) fiber–matrix debonding.

Azwa et al. reported that the water absorbed in polymers is generally either free or bound water [36]. Water molecules that move relatively freely through the microvoids and pores are referred to as free water while those dispersed in the polymer matrix and attached to the polar groups of the polymer are treated as bound water. In a wet environment, water molecules penetrate through microcracks into the composite structure and reduce the interfacial adhesion between the fiber and the matrix. This causes the fibers to swell, which can cause microcracks in the matrix and eventually lead to debonding of the fibers from the matrix [36,37]. The changes that occur in the composites during the immersion test are schematically presented in Figure 5.

Upon adsorption, water creates expansion stress in the polymer and polymer composite. The particles diffuse between the chains of polymer macromolecules and fill the free volume, which increases the distance between the macromolecules and, consequently, causes composite swelling.

The water diffusion in the polymeric materials can induce not only physical phenomena but also certain chemical reactions. The hydrolysis in polymers upon exposure to moisture or water immersion is the most often observed. Its mechanism induces the chain cleavage in the presence of water leading to the formation of two broken chains: A hydrogen ion attached to one extremity and a hydroxide ion to the other one. Generally, hydrolysis is a slow process.

**Figure 6:**
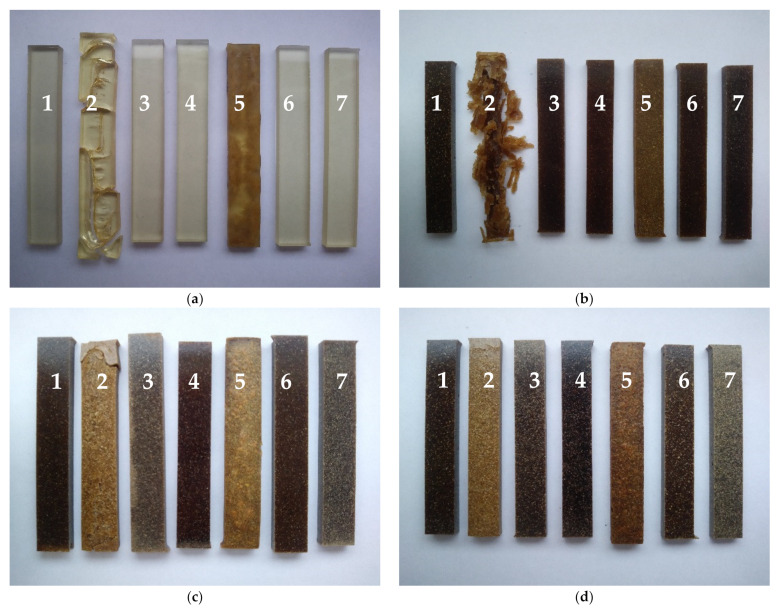
Images of the UPR/PSP composites before and after the immersion test: (**a**) pure UPR; (**b**) UPR + 10; (**c**) UPR + 20; (**d**) UPR + 30. Immersion test liquid standard: 1—reference sample (before test); 2—acetone; 3—distilled water; 4—HCl; 5—NaOH; 6—toluene; 7—Na_2_CO_3_.

**Figure 7:**
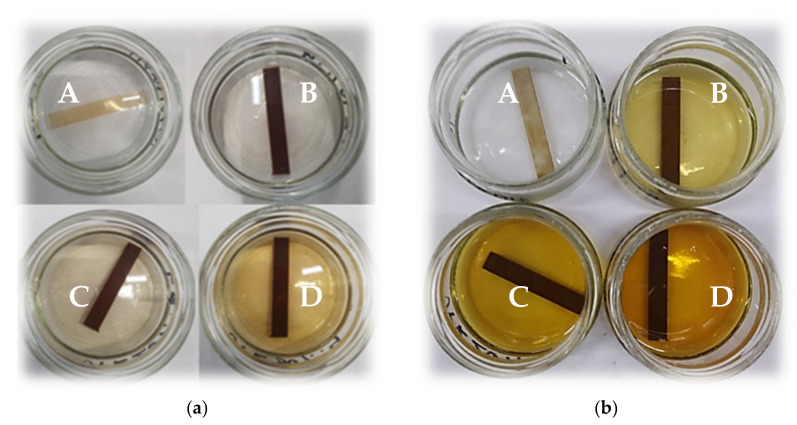
Images of the UPR/PSP composites during the immersion test. The color change of alkaline aqueous solution after: (**a**) 7 days; (**b**) 140 days. A—pure UPR; B—UPR + 10; C—UPR + 20; D—UPR + 30.

After the UPR/PSP composite immersion test, many changes were observed not only in the materials themselves but also in some of the liquid chemicals in which they were immersed (Figure 6 and Figure 7). Certain samples in water and an acid or base were lighter in color, suggesting bleaching.

No significant changes were observed in the hydrochloric acid environment, for the pure resin nor for the composites.

The strong influence of immersion on the behavior of the composites was observed for acetone. The solvent contains a polar hydrophilic group (carbonyl, C=O) that will increase dissolution. For the pure resin, after one day of the immersion test, there was a strong shrinkage, which caused the specimen to crack. Next, the sample underwent successive stages of breaking into smaller and smaller pieces. It was also noticed that the surface became quite sticky. The addition of 10% peanut shell powder meant that such strong shrinkage was not observed, but the delamination process took place. At the end of the test, there was solid core and sheet-like parts remaining.

The alkaline aqueous solution caused the destruction of the polymer matrix as well as changes is the peanut shell powder such as delignification. Although no shrinkage was observed as in the case of acetone, the samples swelled considerably, and the surface was wrinkled and showed that some compounds had leached (Sample 5 in Figure 6). In this environment, aggressive hydrolysis took place. The rate of hydrolysis in the alkaline environment resulted from the formation of a carboxylate anion [38].

The greatest color changes of liquid chemicals were observed for the NaOH solution (Figure 7). Even after 140 days, no color change was observed for the solution in which the pure resin was immersed. In the case of the UPR/PSP composites, along with the amount of filler and the duration of the immersion test, the solution acquired a more intense color. Eventually, it became a yellowish, orange–yellowish, and orange–brown solution, which involved the bleaching and leaching of more and more compounds, such as lignin, present in the biofiller.

The color changes of the other solvents were rather insignificant. Acetone became a very lightly colored, clear solution with a yellow glow. In the case of the samples with an increasing amount of peanut shell powder, the distilled water became cloudy.

**Figure 8:**
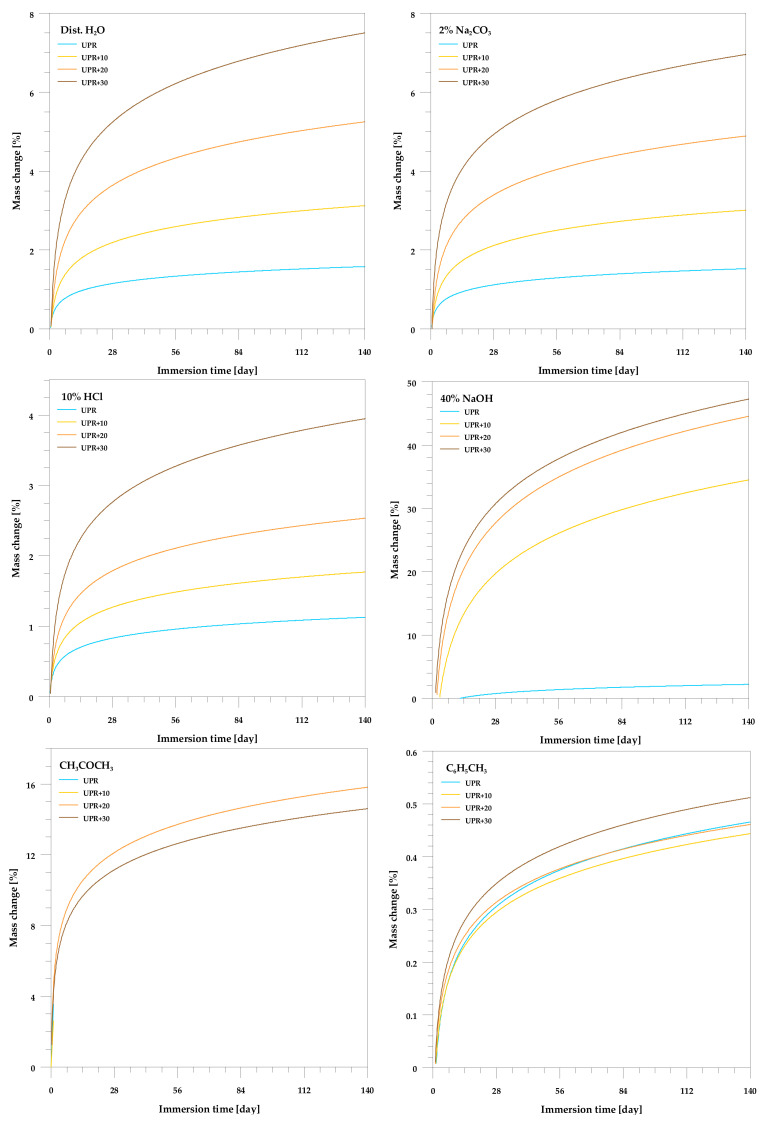
Effect of the chemical resistance of the UPR/PSP composites.

Figure 8 shows the relationships between the mass of the studied composites and their immersion time. Except for acetone, the resin itself hardly changes weight even after 140 days of immersion. For this solvent, partial delamination and exfoliation appeared after just one day (Sample 2 in Figure 6b). The smallest mass changes for both the resin and the composites were found in toluene. In general, all composite samples are characterized by similar behavior in the aqueous solutions, starting with an initial rapid absorption followed by a gradual increase until equilibrium is reached. At any of the similar time intervals, composites containing higher filler loading exhibited larger water absorption.

This was expected due to the hydrophilic nature of the lignocellulosic filler. As a result of the increase in the filler content, hydrogen bonds were formed between the filler hydroxyl groups and the water molecules, which resulted in greater water uptake.

The greatest weight increase can be seen in NaOH. As it was mentioned earlier, sodium hydroxide causes delignification and bleaching of the UPR/PSP composites (see Figure 6 and Figure 7). Certain lignin, wax, and oils that protect the outer surface of the biofiller cell wall are removed, short crystallites are exposed, while cellulose is depolymerized.

**Figure 9:**
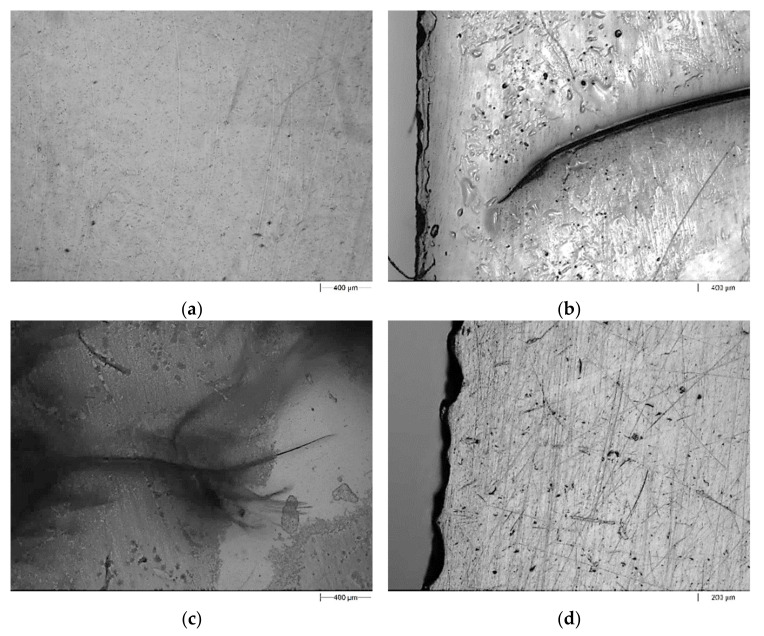
Morphologi 3G microscope images of the pure UPR before (**a**) and after (**b**–**d**) the immersion test. (**a**) Reference sample; (**b**) CH_3_COCH_3_; (**c**) NaOH; (**d**) Na_2_CO_3_.

**Figure 10:**
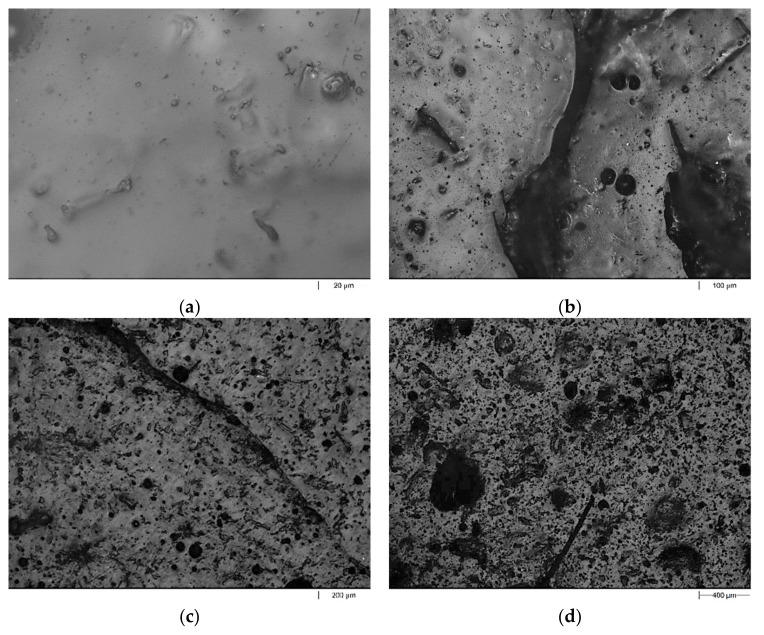
Morphologi 3G microscope images of the UPR + 30 composite before (**a**) and after (**b**–**d**) the immersion test. (**a**) Reference sample; (**b**) CH_3_COCH_3_; (**c**) NaOH; (**d**) Na_2_CO_3_.

The exemplary images of the fracture surface of pure UPR and the UPR/PSP composite containing 30 wt.% peanut shell powder are presented in Figure 9 and Figure 10, respectively. The images of pure UPR (Figure 9a) reveal clear river lines with a smooth fracture surface [27]. Before the immersion test, all composites possessed smooth surface morphology. In the case of the biocomposite (UPR + 30), there are pits in the places where the filler occurs, which could be caused by its swelling with solvents followed by the leaching process. This phenomenon is particularly visible for the composite immersed in acetone. The surface morphology of the sample with the 30% weight fraction of biofiller presented in Figure 10a exhibits quite good interfacial adhesion.

The effect of the immersion test on the surface gloss is presented in Table 4. Gloss is an important parameter characterizing the material surface. The 60° geometry can be used for all materials, but in the case of very high gloss, the measuring method with the 20° geometry is recommended [31].

Before immersion, one can see that the gloss decreased with the addition of the PSP. The samples with up to 20% filler are treated as high-gloss materials as their values at the 60° geometry were greater than 70 GU. For the composite containing 30% of the filler, this value is equal to 62.1 ± 1.7 GU.

The UPR-based composites immersed in toluene with varying amounts of PSP showed similar gloss, varying by 3–5 GU compared to the reference samples. In this case, it was found that neither the filler nor the resin degraded in any way. A difference in the behavior of the composites was observed for acetone. For the pure resin, as already mentioned, after one day of immersion testing, strong shrinkage occurred, which caused the sample to crack. The addition of 10% peanut shell powder resulted in the delamination process. It was not possible to determine the gloss for these samples.

Water absorption also changes the gloss, which is mainly caused by filler swelling. The change in the gloss is noticeable not only for the samples immersed in water but also in the aqueous solutions of HCl, NaOH, and Na_2_CO_3_. As expected, the greatest changes in the gloss value were observed for the composites in contact with the alkaline solvent. Although no shrinkage with acetone was observed, the specimens swelled considerably, whereby the surface was wrinkled and showed certain compounds that were leached. A water solution with salt also had a negative effect on the change in gloss. Both the resin and its composites are not alkali resistant.

The analysis of variance showed that values of gloss for samples with differential percentages of PSP before and after immersion were not statistically significant (at 20° geometry and for all samples immersed in 40% NaOH and acetone). It was confirmed using a one-way analysis of variance (ANOVA) at the significance level *p* < 0.05.

**Figure 11:**
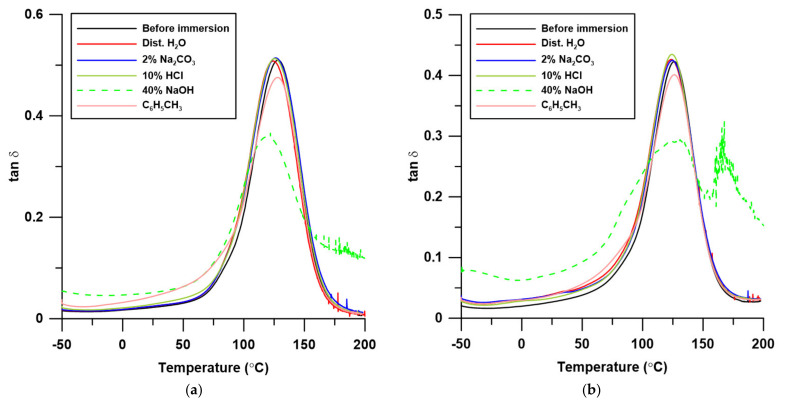

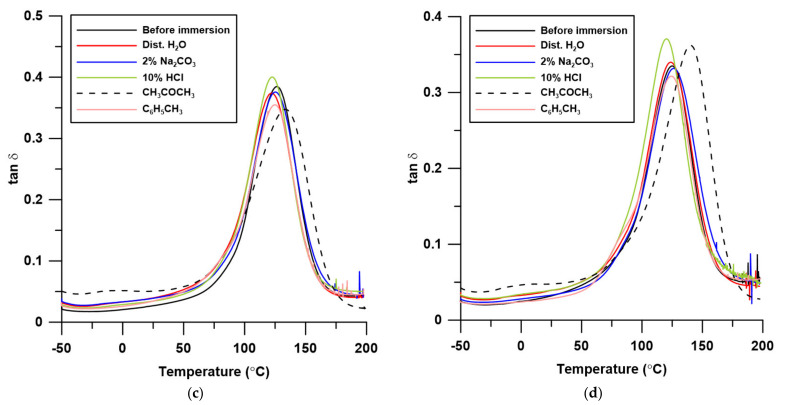
The temperature-dependent graph of damping factor (tan δ): (**a**) Pure UPR; (**b**) UPR + 10; (**c**) UPR + 20; (**d**) UPR + 30.

In Table 5, the numerical data of thermomechanical studies of these composites before and after the immersion test are presented, while Figure 11 shows the curves for the dependence of the damping factor on the temperature. The samples that were not damaged or distorted during the immersion were analyzed.

As can be seen from the data in Table 5, the immersion process influenced changes in the thermomechanical properties of the resin and the corresponding composites, and in many cases, these changes were not significant. In general, in all series, the values of the storage modulus and the glass transition temperatures of the samples after immersion were lower than for the un-immersed samples. On the other hand, the FWHM values for composites with 20 and 30% filler content clearly increased (except for UPR + 20 and UPR + 30 immersed in an acidic environment). This shows that the homogeneity of the samples was reduced during the immersion process. The damping values (tan δ_max_) of the resin and composites did not change significantly, but for the samples conditioned in the acid environment, the damping clearly decreased, while for the samples immersed in toluene, it increased.

The changes in thermomechanical properties in an alkaline environment may result from the alkaline hydrolysis of ester bonds contained in the resin, and thus the degradation of polymer chains. In the case of toluene, it may dissolve the styrene fragments of the resin. A similar observation was made by Guhanathan and Saroja Devi [39]. For polyester immersion in alkali, all mechanical properties (tensile strength, tensile modulus, flexural strength, flexural modulus, compressive strength, impact strength, and hardness) deteriorated drastically. This may be attributed to the hydrolysis of the ester linkages in the polyester matrix, leading to degradation in the polymer chains and a decrease in the molecular weight, together with hydrolysis of the interfacial bonds.

## 4. Conclusions

Composites of different loadings of PSP and unsaturated polyester resin were obtained and investigated in view of their structural, thermal, thermomechanical, and chemoresistant properties. The results show that the commercially available resin based on recycled PET can be used for the preparation of homogeneous composites with PSP up to 30%. Although the initial decomposition temperature of composites is much lower than the decomposition temperature of pure resin, the remaining thermal properties are similar. The gloss values indicate that both the pure resin and its composites belong to the group of high-gloss materials, while after the test, only resin and the 10% composites, which were immersed in water, HCl, and toluene, exhibited gloss.

The changes in the properties of pure resin and composites after immersion are dependent on the chemical nature of the solvent in which they were immersed. Both the resin and its composites are not resistant to acetone, as it caused the destruction of the polymer matrix as well as changes in the peanut shell powder, such as delignification. After one day of immersion, a strong shrinkage effect was observed in acetone leading to the specimen cracking. The changes in thermomechanical properties in 40% NaOH are particularly visible for the pure resin and its composite with 10% PSP, which means that in an alkaline environment, hydrolysis of the ester bonds contained in the resin takes place. Moreover, the effect of water on the UPR/PSP composites should not be underestimated, as when water molecules penetrate the polymer composite materials, a plasticizing effect is observed.

This does not change the fact that peanut shell powder is available and tends to be processed with different types of polymer matrices. These composites are a potential alternative material that can be used in the household, especially in packaging, automotive, and construction applications, due to their low weight and low cost [40].

## Figures and Tables

**Table 1:** Formulation of UPR/PSP composites.

Sample	Composition (Weight Proportion)	
	UPR ^1^ [wt. %]	PSP ^2^ [wt. %]
pure UPR	100	0
UPR + 10	90	10
UPR + 20	80	20
UPR + 30	70	30

^1^ UPR—unsaturated polyester resin; ^2^ PSP—peanut shell powder.

**Table 2:** Characteristic FT-IR bands of peanut shell powder.

Wavenumber [cm^−1^]	Band Assignment	Possible Compound
3340	OH stretching; alcohols, phenols, acids	cellulose, hemicellulose, lignin
2928, 2854	C-H stretching; methyl, methylene	cellulose, hemicellulose, lignin
1738	C=O stretching;	hemicellulose, lignin
1636	C=O stretching;	lignin
1609	aromatic skeletal, C=O stretching, absorbed OH	hemicellulose, lignin
1510	C=C-C aromatic ring stretching	lignin
1449	C-H deformation; methyl, methylene	lignin
1422	CH_2_ bending, C=O stretching, CH deformation	cellulose, hemicellulose, lignin
1369	CH bending, CH stretching in CH_3_	cellulose, hemicellulose, lignin
1318	CH_2_ wagging, C-O stretching of substituted aromatic units	cellulose, hemicellulose, lignin
1261	syringal ring breathing, C-O stretching	lignin, xylan
1235	C-O stretching of guaiacyl unit	lignin
1150	C-O-C stretching	cellulose, hemicellulose
1100	aromatic C-H in plane deformation	lignin
1058	C-OH stretching, C-O deformation	cellulose, hemicellulose, lignin
1028	C-O stretching, aromatic C-H in plane deformation	cellulose, lignin
897	CH deformation of glucose ring	cellulose, hemicellulose
667	β-glycosidic ether ethyl linkage	cellulose, hemicellulose

**Table 3:** Thermogravimetric analysis data for the UPR/PSP composites.

Sample	$T_{1\%}$^1^ [°C]	$T_{5\%}{\;}^{2}\;[{}^{\circ}C]$	$T_{10\%}{\;}^{3}\;[{}^{\circ}C]$	$T_{50\%}{\;}^{4}\;[{}^{\circ}C]$	$T_{\max}{\;}^{5}\;[{}^{\circ}C]$	$MC{\;}^{6}\;[\%]$	$RM{\;}^{7}\;[\%]$
PSP	48	89	225	296	61; 295; 381; 430	−5.99; −53.37; −24.46; −12.10	3.72
pure UPR	167	299	336	392	394; 519	−85.71; −14.19	---
UPR + 10	163	286	324	385	391; 485	−82.17; −18.56	0.65
UPR + 20	131	269	315	390	390; 480	−77.12; −21.89	0.97
UPR + 30	142	266	307	388	386; 478	−75.42; −23.52	1.22

^1^ Temperature of 1% mass loss; ^2^ Temperature of 5% mass loss; ^3^ Temperature of 10% mass loss; ^4^ Temperature of 50% mass loss; ^5^ Maximum decomposition temperature; ^6^ Mass Change; ^7^ Residual Mass.

**Table 4:** Gloss measurement data of the UPR/PSP composites before and after the immersion test (mean ± SD; *n* = 10).

Sample	Liquid Test Standard	Gloss [GU]					
		20°		60°		85°	
		Before	After	Before	After	Before	After
pure UPR	Dist. H_2_O	111.9 ± 0.2 ^a^	103.4 ± 0.3 ^a^	121.3 ± 0.3 ^b^	114.8 ± 0.2 ^b^	100.3 ± 0.2 ^b^	101.3 ± 0.2 ^b^
	2% Na_2_CO_3_		53.1 ± 0.2 ^a^		88.5 ± 01 ^b^		100.2 ± 0.2 ^b^
	10% HCl		106.5 ± 0.1 ^a^		115.3 ± 0.2 ^b^		99.0 ± 0.1 ^b^
	40% NaOH		9.3 ± 0.1 ^a^		41.1 ± 1.3 ^a^		61.5 ± 0.1 ^a^
	CH_3_COCH_3_		- *		- *		- *
	C_6_H_5_CH_3_		107.9 ± 0.2 ^a^		118.3 ± 1.3 ^b^		99.5 ± 0.2 ^b^
UPR + 10	Dist. H_2_O	53.6 ± 0.1 ^a^	37.9 ± 0.2 ^a^	81.4 ± 0.1 ^b^	71.0 ± 0.2 ^b^	90.1 ± 0.1 ^b^	84.7 ± 0.2 ^b^
	2% Na_2_CO_3_		27.9 ± 0.3 ^a^		61.2 ± 0.4 ^b^		86.0 ± 0.1 ^b^
	10% HCl		36.4 ± 1.2 ^a^		72.5 ± 0.2 ^b^		84.2 ± 0.1 ^b^
	40% NaOH		0.4 ± 0.2 ^a^		8.7 ± 1.2 ^a^		13.9 ± 1.6 ^a^
	CH_3_COCH_3_		- *		- *		- *
	C_6_H_5_CH_3_		45.1 ± 1.1 ^a^		78.4 ± 0.7 ^b^		89.6 ± 0.4 ^b^
UPR + 20	Dist. H_2_O	37.6 ± 1.6 ^a^	18.1 ± 1.2 ^a^	72.3 ± 0.2 ^b^	52.7 ± 0.1 ^b^	85.9 ± 0.2	66.9 ± 1.2 ^b^
	2% Na_2_CO_3_		10.6 ± 0.8 ^a^		39.4 ± 1.3 ^b^		59.9 ± 0.6 ^b^
	10% HCl		16.5 ± 1.2 ^a^		53.9 ± 0.8 ^b^		69.6 ± 0.5 ^b^
	40% NaOH		0.3 ± 0.1 ^a^		5.1 ± 1.2 ^a^		8.5 ± 1.7 ^a^
	CH_3_COCH_3_		8.6 ± 0.4 ^a^		46.0 ± 1.6 ^a^		57.7 ± 1.1 ^a^
	C_6_H_5_CH_3_		29.9 ± 1.7 ^a^		67.3 ± 0.8 ^b^		82.4 ± 1.7 ^b^
UPR + 30	Dist. H_2_O	24.5 ± 1.2 ^a^	12.9 ± 0.2 ^a^	62.1 ± 1.7 ^b^	47.3 ± 0.2 ^b^	78.3 ± 0.3	61.5 ± 1.5 ^b^
	2% Na_2_CO_3_		7.0 ± 1.2 ^a^		31.4 ± 1.6 ^b^		50.3 ± 0.7 ^b^
	10% HCl		11.5 ± 0.8 ^a^		48.2 ± 0.9 ^b^		62.7 ± 0.5 ^b^
	40% NaOH		0.2 ± 0.1 ^a^		4.2 ± 0.5 ^a^		5.8 ± 1.1 ^a^
	CH_3_COCH_3_		7.0 ± 0.9 ^a^		35.3 ± 1.3 ^a^		50.8 ± 0.2 ^a^
	C_6_H_5_CH_3_		16.7 ± 1.5 ^a^		58.0 ± 0.7 ^b^		75.9 ± 1.4 ^b^

^a^ Statistically not significant, ^b^ Statistically significant. * The sample was damaged during the immersion test—no gloss measurement possible.

**Table 5:** Thermomechanical data for the UPR/PSP composites before and after the immersion test.

Sample	Liquid Test Standard	$E^{\prime }$ ^1^				$T_{g}\;[{}^{\circ}C]{\;}^{2}$		$tan\delta_{max}{\;}^{3}$		$FWHM\;[{}^{\circ}C]{\;}^{4}$	
		$E^{\prime }$ (20 °C) [GPa]		$E^{\prime }$ (180 °C) [MPa]		From tan δ					
		Before	After	Before	After	Before	After	Before	After	Before	After
pure UPR	Dist. H_2_O	3.052	2.921	24.37	19.53	128.33	124.53	0.497	0.498	44.29	45.92
	2% Na_2_CO_3_		3.011		16.00		126.26		0.501		47.63
	10% HCl		3.135		17.17		125.50		0.498		47.18
	40% NaOH		2.055		51.62		118.28		0.258		47.99
	CH_3_COCH_3_		-		-		-		-		-
	C_6_H_5_CH_3_		2.870		16.76		128.15		0.461		46.59
UPR + 10	Dist. H_2_O	3.301	2.961	47.78	39.13	126.14	123.73	0.399	0.398	40.19	42.95
	2% Na_2_CO_3_		3.033		40.78		124.54		0.395		41.98
	10% HCl		3.156		39.39		124.10		0.407		41.70
	40% NaOH		-		-		-		-		-
	CH_3_COCH_3_		-		-		-		-		-
	C_6_H_5_CH_3_		3.103		38.21		126.23		0.372		41.93
UPR + 20	Dist. H_2_O	3.345	2.858	72.69	62.99	126.42	122.11	0.349	0.338	40.74	42.86
	2% Na_2_CO_3_		2.828		59.45		125.32		0.336		42.84
	10% HCl		3.064		52.95		122.41		0.359		39.41
	40% NaOH		-		-		-		-		-
	CH_3_COCH_3_		1.435		46.82		133.67		0.315		51.74
	C_6_H_5_CH_3_		3.235		61.41		124.91		0.317		41.59
UPR + 30	Dist. H_2_O	3.636	2.836	107.98	87.34	125.01	123.82	0.293	0.300	40.92	43.41
	2% Na_2_CO_3_		2.747		84.87		126.37		0.290		44.70
	10% HCl		2.647		55.22		120.22		0.329		39.99
	40% NaOH		-		-		-		-		-
	CH_3_COCH_3_		1.703		61.08		140.23		0.328		45.70
	C_6_H_5_CH_3_		3.164		89.42		124.31		0.278		42.31

^1^ Storage Modulus, Glassy and Rubbery; ^2^ Glass-Transition Temperature; ^3^ Mechanical Loss Factor; ^4^ Full Width at Half Maximum.

## Data Availability

Not applicable.
